# Odontogenic tumors: A retrospective study of four Brazilian diagnostic pathology centers

**DOI:** 10.4317/medoral.17630

**Published:** 2011-12-06

**Authors:** Daniela O P. da-Costa, Almir S. Maurício, Paulo A S. de-Faria, Licínio E. da-Silva, Adalberto Mosqueda-Taylor, Simone Q C. Lourenço

**Affiliations:** 1 MD. PhD Pathology Graduate Program, Fluminense Federal University (UFF), Brazil; 2 DC Anatomic Pathology Service, Army Central Hospital (HCE-RJ), Brazil; 3 MD Pathology Division, National Cancer Institute (INCA), Brazil; 4 MD Department of Statistics, Fluminense Federal University (UFF), Brazil; 5 DDS, MSc Department of Health Care, Universidad Autónoma Metropolitana Xochimilco, México

## Abstract

Objective: This article presents the results of a retrospective study of the frequency and classification of odontogenic tumors recorded at four centers of diagnostic pathology in Rio de Janeiro, Brazil. Study Design: All medical records and microscopic slides of odontogenic tumor specimens for the years 1997 to 2007 were retrieved from the files of four services of diagnostic pathology in Rio de Janeiro City. Diagnoses were re-evaluated and the tumors classified according to the latest (2005) World Health Organization Classification of Tumors. Results: A total of 201 odontogenic tumors were found among 15,758 oral biopsies (1.3%). The frequencies of these tumors at the four centers ranged from 0.5% at the National Cancer Institute to 3.3% in a private laboratory. Chi-square analysis revealed statistically significant differences (p<0.05) between the proportions of odontogenic tumors in the studied centers. Of these, 94.5% were benign and 5.5% were malignant. Keratocystic odontogenic tumor (32.3%) was the most frequent lesion, followed by ameloblastoma (29.8%) and odontoma (18.4%). Conclusions: Odontogenic tumors are uncommon in Brazil. Different pathology laboratories reported divergent frequencies of odontogenic tumors, which may reflect institutional specializations and the patient populations served.

** Key words:**Odontogenic tumors, jaw neoplasms, epidemiology, WHO classification.

## Introduction

Odontogenic tumors (OT) comprise an infrequent group of lesions arising from the tooth-producing tissues or its remnants, and these range from hamartomatous or non-neoplastic proliferations to benign and malignant neoplasm with variable aggressiveness and metastatic potential (1,2).

The first internationally accepted classification system for these tumors was published in 1971 by the World Health Organization (WHO), which was reviewed and updated in 1992 and in 2005 (2). One of the main modifications found in the newest edition was the addition of the odontogenic keratocyst as a benign, but locally aggressive epithelial odontogenic tumor, which has been renamed as keratocystic odontogenic tumor (KCOT).

The knowledge of the histological features of the different odontogenic tumors, as well as of their clinicopathological features recorded in diverse populations worldwide are important points that may help to identify the groups at risk and possible factors associated to the development of these infrequent, but biologically complicated lesions (2).

Data from the literature show differences in the relative frequencies of these tumors (3-24). Several reports on series of OT from different countries have documented distinct geographic variations in tumor prevalence, with the main difference being the relative incidence of ameloblastoma and odontoma (4,5,8,10,16,19). However, the inclusion of the KCOT has produced an increase in the frequency and prevalence of OT (20).

There is limited information in the literature about the prevalence of OT in Brazil (21-24). The objective of the present study was to establish the frequencies and types of OT diagnosed in four centers of diagnostic pathology in Brazil, using the 2005 WHO histopathological classification of tumors and to compare the results with those found in similar studies from other parts of the world.

## Material and Methods

The histologic material that served as the basis for this study was obtained from the following pathology diagnostic centers located in Rio de Janeiro: National Cancer Institute (INCA), Army Central Hospital (HCA), the Anatomic Pathology Service of the Antonio Pedro University Hospital (Federal Fluminense University - HUAP) and a private pathology laboratory. Case records of patients with OT and KCOT spanning an 11-year period (1997-2007) were used. Inclusion criteria: cases diagnosed histopathologically as OT according to the 2005 W.H.O. Histological Classification of Tumors which had clinicopathologic information. Slides without histopathologic criteria for definitive diagnosis of OT and cases without slides and paraffin-embedded tumor specimens were excluded. Basic clinicopathologic features, including histopathology type, patient’s age, gender, and the tumor site, were obtained from the medical records.

The research protocol was approved by the Research Ethics Committee of Federal Fluminense University (Niterói-RJ), the National Cancer Institute (RJ), and the Army Central Hospital (RJ) for its implementation. The Director of the private laboratory also authorized the research.

Hematoxylin and eosin-stained slides of all the cases were retrieved from the laboratories for review and the diagnoses were reevaluated and the tumors reclassified according to WHO (2005) criteria. The independent opinions of two examiners were compared to reach the final diagnosis and in cases of doubt we consulted another expert oral pathologist to obtain a diagnosis by consensus.

For the statistical analysis, commercially available software (SPSS 10.0; SPSS Inc, Chicago, IL) was used. Descriptive statistics were used in a preliminary analysis of the relationship between baseline variables. Continuous variables were categorized to facilitate data analysis and presentation. Gender and tumor site analyses were done using the binomial test. The chi-square test was used to investigate differences between proportions of independent groups. Tests were considered statistically significant when the p-value was <0.05.

## Results

A total of 15,748 oral biopsies were registered during the 11-year period of this retrospective study, in which we found 288 cases diagnosed as OT. After applying the inclusion criteria and reevaluating the hematoxylin and eosin-stained sections, 87 cases were excluded, leaving a total of 201 cases (1.3%). Of the excluded cases, 46 were rejected because they did not satisfy the WHO criteria and 36 lacked slides/paraffin-embedded tumor specimens or data. One case of orthokeratinized odontogenic cyst, one keratoameloblastoma, one unclassified malignant odontogenic tumor and two adenomatoid odontogenic hamartomas (14) were excluded on the basis of the classification used.

The frequency of OT diagnosed at the four diagnostic centers ranged from 0.5% at the INCA to 3.3% recorded at the private laboratory. Chi-square analysis revealed a statistically significant difference (p<0.05) between the proportions of OT in the centers studied (χ2 = 134.592; d.f. = 3; p<0.0001).The distribution of oral lesions and OT in the four centers are shown in ( Table 1).

**Table 1:**
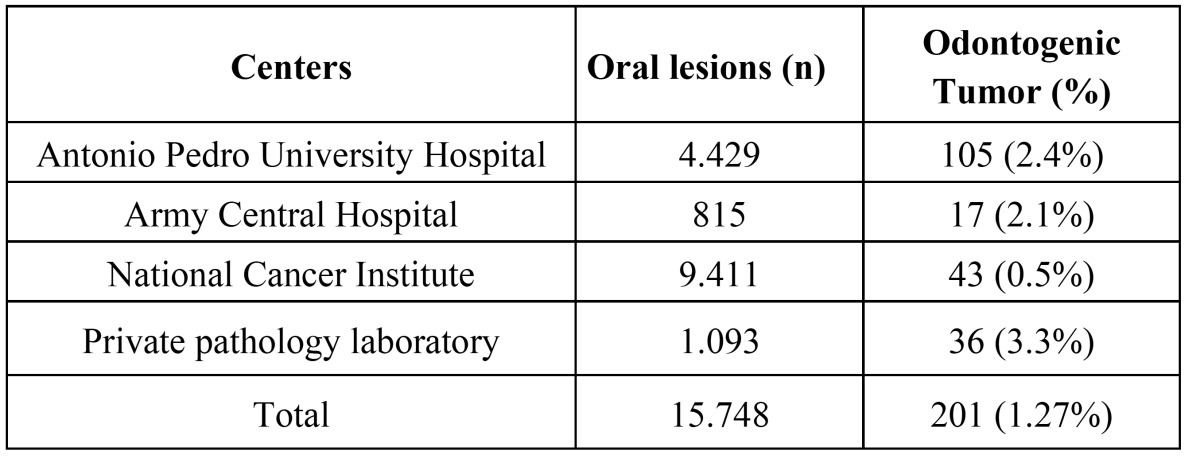
Distribution of oral lesions and odontogenic tumors in the diagnostic pathology centers.

Of the 201 cases of OT, 196 (97.5%) were intraosseous and five (2.5%) were extraosseous (2 peripheral ameloblastomas, 2 peripheral calcifying epithelial odontogenic tumor and one peripheral calcifying cystic odontogenic tumor). There were 190 benign (94.5%) and 11 malignant (5.5%) tumors. The most frequent lesion was KCOT (32.3%), followed by ameloblastoma (29.8%) and odontoma (18.4%). Statistical analysis revealed no significant difference between the proportions of these three groups (p<0.05). Ameloblastic carcinoma was the most common malignant tumor (3.5%). The distribution of histological types and the relative frequencies of OT by centers of diagnosis are shown in ( Table 2).

**Table 2:**
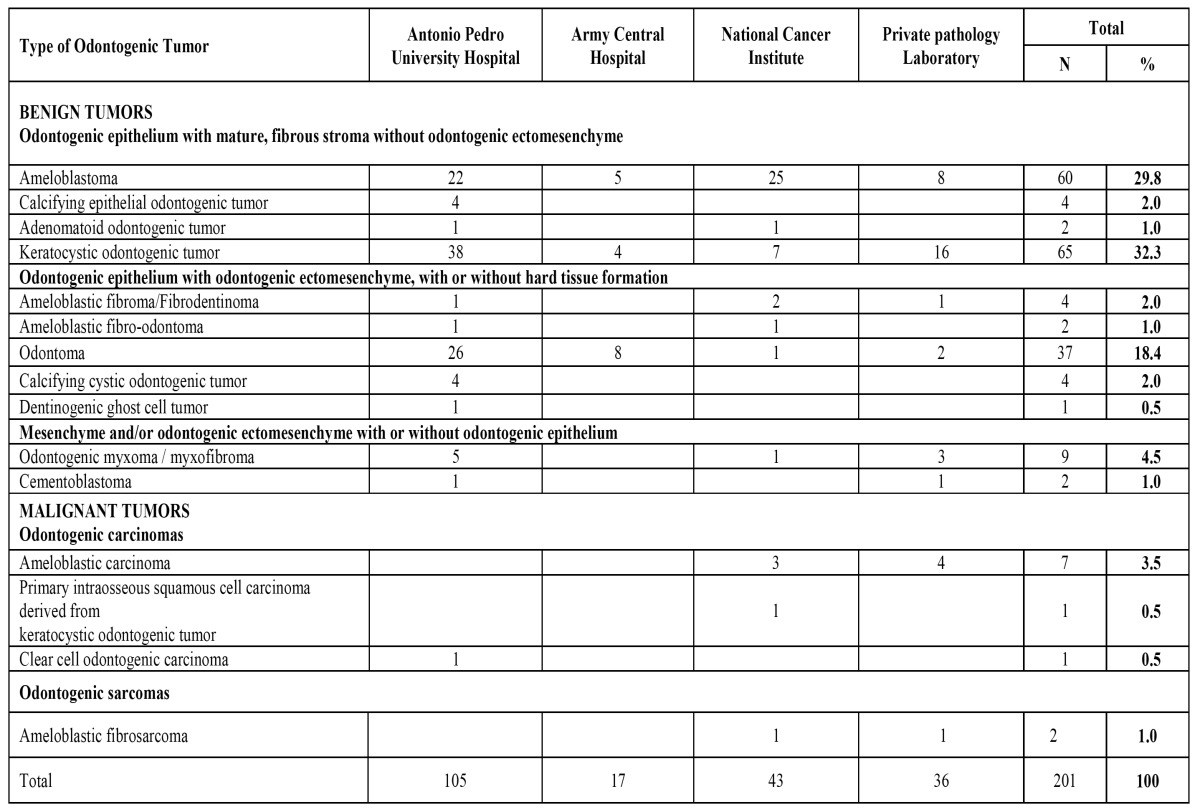
Distribution of 201 benign and malignant odontogenic tumors from four diagnostic pathology centers.

Regarding gender and age, 57.2% of all tumors occurred in males and 42.8% in females. Statistical analysis revealed no significant difference in the distribution of OT in relation to gender (p-value = 0.67, binomial test). The male-female ratio was 1.3:1. The age of patients at diagnosis (where this information was available) ranged from 5 to 82 years, with a mean of 35 years (s.d. = 19.4 years), most often affecting patients in the third and fourth decades.

The mandible (140 cases, 69.6%) was 2.7 times more commonly affected than the maxilla (52 cases; 25.8%), and this was particularly remarkable for ameloblastoma and KCOT (p < 0.05, binomial test). ( Table 3) shows the gender, age and site distribution of 201 odontogenic tumors at the four diagnostic pathology centers.

**Table 3:**
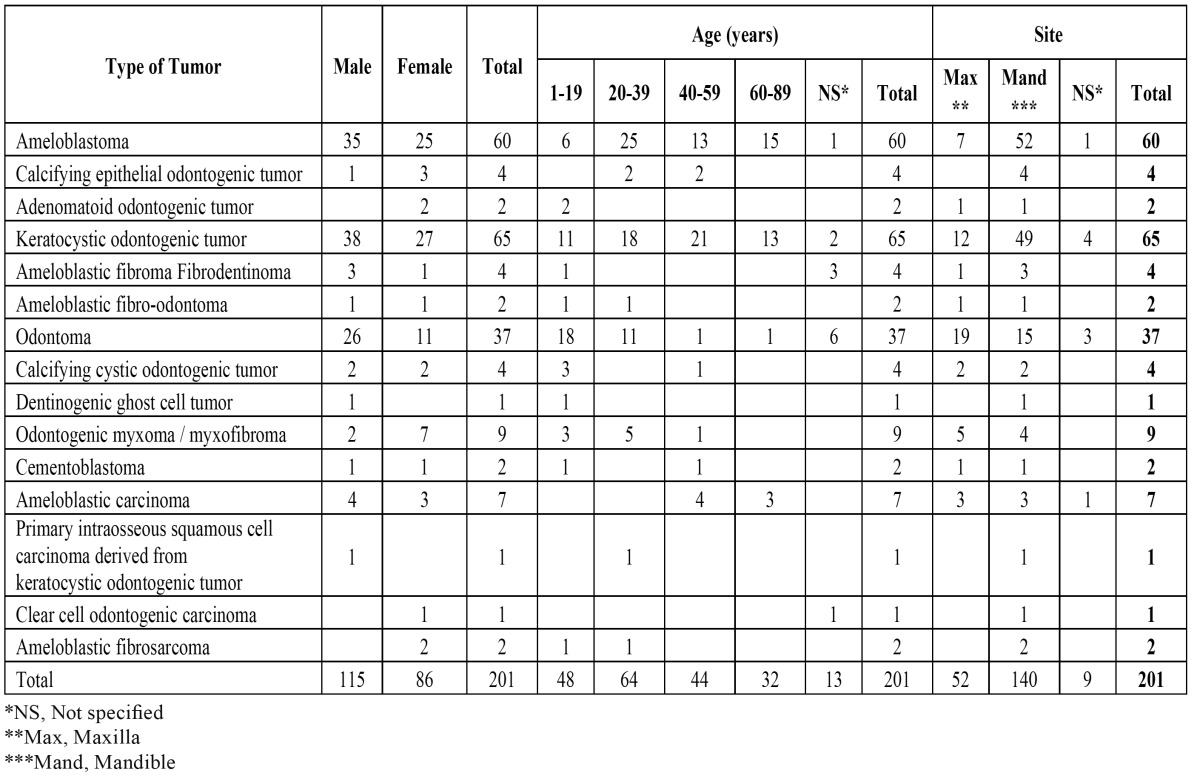
Gender, age and site distribution of 201 odontogenic tumors of patients at four diagnostic pathology centers.

## Discussion

Studies of OT from several parts of the world indicate that knowing the frequency and basic clinical features of these lesions is important because they allow to know more precisely the expression of these lesions in the diverse populations, which in turn help to identify the groups at risk and possible factors associated to their development, as well as to develop more precisely differential diagnoses (2).

Few reports have been published in the English language literature using the 2005 WHO classification of OT (12,16,18,19,20,24). For some authors (2,20), the inclusion of KCOT is a factor that modify the relative frequency of the OT and because it is a relatively common tumor of the jaws, the KCOT will occupy a preponderant place in the prevalence of OT in epidemiological studies of this group of lesions.

The relative frequency of odontogenic tumors in the present study was 1.3% of all specimens recorded between January 1997 and December 2007. This figure is similar to what has been reported in other studies, as they represent less than 3% of oral and maxillofacial specimens studied in North American (3,5), South American (7,21-24) and Europen series (8,11). On the other hand, in Africa and Asia OT comprise from 3.9% to 9.6% of all oral lesions (9,15,16), although an Iranian series had a frequency of 1.9% (18).

This study confirms that benign tumors (94.5%) are the most frequently seen OT; however, the malignant OT represented 5.5% in the present series. This frequency of malignant tumors is only similar to those reported in China (6,16), but it is higher than that those published in most other series (3,4,5,7,10,21-24).

As expected, we found KCOT to be the most common OT (31.8%), followed by ameloblastoma (30.3%) and odontoma (18.4%). There were no statistically significant differences between the ameloblastoma and KCOT and odontoma groups. These results are comparable with the corresponding data reported by other authors that followed the 2005 W.H.O. Histological Classification of Tumors (16). In contrast, Jing et al. (12), Tawfik and Zyada (19) and Osterne et al. (24) reported ameloblastoma to be most frequent lesion in Chinese, Egyptian and Brazilian populations, followed by KCOT and odontoma. In accordance with Tawfik and Zyada (19), these discrepancies probably result from geographic variation and underestimated cases of odontoma.

According to most studies, the main difference reported in the literature is related to the relative frequency of ameloblastoma and odontoma. In China (6,12,16), Egypt (19), Sri Lanka (13), Brazil (21,23) and in certain countries of Africa (8,9), the frequency of ameloblastoma was higher than odontoma, in contrast to what has been observed in some countries of the American Continent (3,5,7,21). Jing et al. (12) and Tawfik and Zyada (19) considered that the low number of odontoma cases recorded in their study was due to the fact that these lesions have a limited growth, are often asymptomatic and diagnosed only radiographically (left untreated or without pathologic examination). Also, they may go undiagnosed in patients who do not receive regular dental check-ups (19). These results show that studies in teaching institutions and private hospitals equipped with orthopantomograms do not underestimate cases of odontomas.

Simon et al. (10) argued that the relatively high frequency of odontogenic tumors in many African countries stems from unavailability of facilities for proper investigations of non-aggressive tumors and delayed diagnosis at primary centers. Thus, retrospective studies in African hospitals usually report larger numbers of ameloblastomas. Therefore, as has been pointed out in previous studies, the relatively high frequency of malignant tumors observed in this and some other studies may be a reflection of the types of pathology services surveyed (5,22).

In relation to sociodemographic data, a higher proportion of males was affected with OT and the average age at diagnosis was 35 years, which is in agreement with the results of Simon et al. (10) in Tanzania. However, there are some other studies that have reported differences associated with this gender and age distribution of OT (7,21). The majority of articles confirm the mandible as the anatomic site most frequently affected by OT, especially by ameloblastoma and KCOT, which agrees with our findings (5,17,19,22,24.

Our results show that the proportion of OT varied with respect to the total number of oral lesions recorded at the four institutions studied, with percentages ranging from 0.5% to 3.3%. The private pathology laboratory identified the highest proportion of OT and INCA the lowest. The results of this study are in agreement with those of Daley et al. (3), who consider that data from a diagnostic biopsy service are biased in several respects.

The private laboratory showed a greater frequency of OT and oral lesions than the other laboratories, possibly because this center is specialized in oral pathology, whereas INCA is the primary cancer referral center for Brazil, seeing a large number of patients with oral lesions but predominantly with malignancies such as oral squamous cell carcinomas. Regarding OT, HUAP had the greatest number of cases (n=105). This could be because this center has been an oral pathology service in a University Hospital for many years and offers diagnostic services to the general public. A low number of cases of oral lesions and OT were observed at HCA. This may be explained by the fact that HCA is a military institution which serves a narrow population base.

Mosqueda-Taylor et al. (5), reported the relative frequencies of OT in different centers of diagnosis in Mexico, and their results are very similar to ours. The highest frequency of OT (3.7%) in their series was observed in a private oral pathology laboratory. Two universities in their study had equal values (2.5%) and the National Institute of Cancerology (Mexico) reported a frequency of OT of 0.8%, similar to the frequency at HUAP (2.4%) and INCA (0.5%) seen in the present study.

Published studies regarding the frequency and type of OT suggest that geographical variation, deficiency in dental care, as well as underestimation of cases, especially of odontoma, are factors that influence the results. Our study shows that OT are uncommon in Brazil and that the frequency of these tumors vary in accordance with the type of diagnostic center, even within the same geographical region.
